# A Degenerative Retinal Process in HIV-Associated Non-Infectious Retinopathy

**DOI:** 10.1371/journal.pone.0074712

**Published:** 2013-09-17

**Authors:** Igor Kozak, Roman Sasik, William R. Freeman, L. James Sprague, Maria Laura Gomez, Lingyun Cheng, Sharif El-Emam, Francesca Mojana, Dirk-Uwe Bartsch, Jenny Bosten, Radha Ayyagari, Gary Hardiman

**Affiliations:** 1 Jacobs Retina Center at the Shiley Eye Center, University of California San Diego, Department of Ophthalmology, La Jolla, California, United States of America; 2 University of California San Diego, Department of Medicine, La Jolla, California, United States of America; 3 BIOGEM, University of California San Diego, School of Medicine, La Jolla, California, United States of America; 4 University of California San Diego, Department of Psychology, La Jolla, California, United States of America; 5 Computational Science Research Center and Biomedical Informatics Research Center San Diego State University, San Diego, California, United States of America; National Eye Institute, United States of America

## Abstract

HIV retinopathy is the most common non-infectious complication in the eyes of HIV-positive individuals. Oncotic lesions in the retinal nerve fiber layer, referred to as cotton wool spots (CWS), and intraretinal (IR) hemorrhages are frequently observed but are not unique to this pathology. HIV-positive patients have impaired color vision and contrast sensitivity, which worsens with age. Evidence of inner–retinal lesions and damage have been documented ophthalmoscopically, however their long term structural effect has not been investigated. It has been hypothesized that they may be partially responsible for loss of visual function and visual field. In this study we utilized clinical data, retinal imaging and transcriptomics approaches to comprehensively interrogate non-infectious HIV retinopathy. The methods employed encompassed clinical examinations, fundus photography, indirect ophthalmoscopy, Farmsworth-Munsell 100 hue discrimination testing and Illumina BeadChip analyses. Here we show that changes in the outer retina, specifically in the retinal pigment epithelium (RPE) and photoreceptor outer segments (POS) contribute to vision changes in non-infectious HIV retinopathy. We find that in HIV-positive retinae there is an induction of rhodopsin and other transcripts (including *PDE6A*, *PDE6B*, *PDE6G*, *CNGA1, CNGB1*, *CRX*, *NRL*) involved in visual transduction, as well as structural components of the rod photoreceptors (*ABCA4* and *ROM1*). This is consistent with an increased rate of renewal of rod outer segments induced via increased phagocytosis by HIV-infected RPE previously reported in culture. Cone-specific transcripts (*OPN1SW*, *OPN1LW*, *PDE6C, PDE6H* and *GRK7*) are uniformly downregulated in HIV positive retina, likely due to a partial loss of cone photoreceptors. Active cotton wool spots and intraretinal hemorrhages (IRH) may not affect photoreceptors directly and the interaction of photoreceptors with the aging RPE may be the key to the progressive vision changes in HIV-positive patients.

## Introduction

Human immunodeficiency virus (HIV) remains a major public health problem in the United States with 56,000 new HIV infections per year [1]. The current prevalence of HIV infection in the United States is estimated to be over one million with a disproportionate distribution among minorities, and a reemerging HIV/AIDS epidemic in gay men [2-4]. Worldwide the HIV pandemic continues with 33 million infected individuals and an annual infection rate of 2.7 million with 2 million deaths. Of those infected less than half of those who urgently needed antiretroviral therapy (ART) to prevent serious illness or death were actually receiving it [5]. The increased life expectancy of human immunodeficiency virus (HIV) positive patients has led to evidence of complications not directly related to opportunistic infections [6].

The human retina, an extension of the central nervous system, is composed of five major neuronal types and one glial cell type originating from the same pool of progenitor cells. HIV retinopathy with vision abnormalities is the most common non-infectious complication in non-immunocompromised HIV-positive individuals [7]. It is an ischemic retinopathy. Oncotic lesions in the retinal nerve fiber layer, also commonly referred to as cotton wool spots (CWS), and intraretinal (IR) hemorrhages are commonly observed but are not unique to this pathology [8-13]. Cotton wool spots manifest as fluffy white patches on the retina and represent an abnormal finding upon funduscopic examination of the retina. CWS result from nerve fiber damage and accumulation of axoplasmic material within the nerve fiber layer [14]. They occur in approximately 50-60% of patients with advanced HIV disease and are the earliest and most consistent finding in HIV retinopathy [15].

Evidence of inner–retinal lesions and damage has been documented ophthalmoscopically; however their long-term structural effect has not been investigated. It has been hypothesized that they may be partially responsible for loss of visual function and visual field [16-18]. A limited number of human studies have been performed with animal models providing the bulk of our knowledge on retinal gene expression to date. Previous molecular studies of HIV retinopathy have been confined to a selected group of molecules or genes and have not provided a systems-level view [19].

In this report we combine clinical data, retinal imaging and transcriptomics data on non-infectious HIV retinopathy. Recent advances in retinal imaging provide comprehensive *in vivo* histology allowing detection of prior retinal insults from HIV disease on a scale that was not possible a few years ago. Furthermore we investigate using systems-level analysis retinal tissue in autopsy eyes obtained from HIV-positive donors with either no visible clinical pathology on gross examination or intraretinal hemorrhages characteristic of HIV retinopathy. We demonstrate long-term structural and functional modifications of the retina caused by HIV retinopathy in the absence of infection or clinically apparent lesions.

## Results and Discussion

### CWS, after resolution leave behind permanent damage to the inner retinal layer

**Figure 1:**
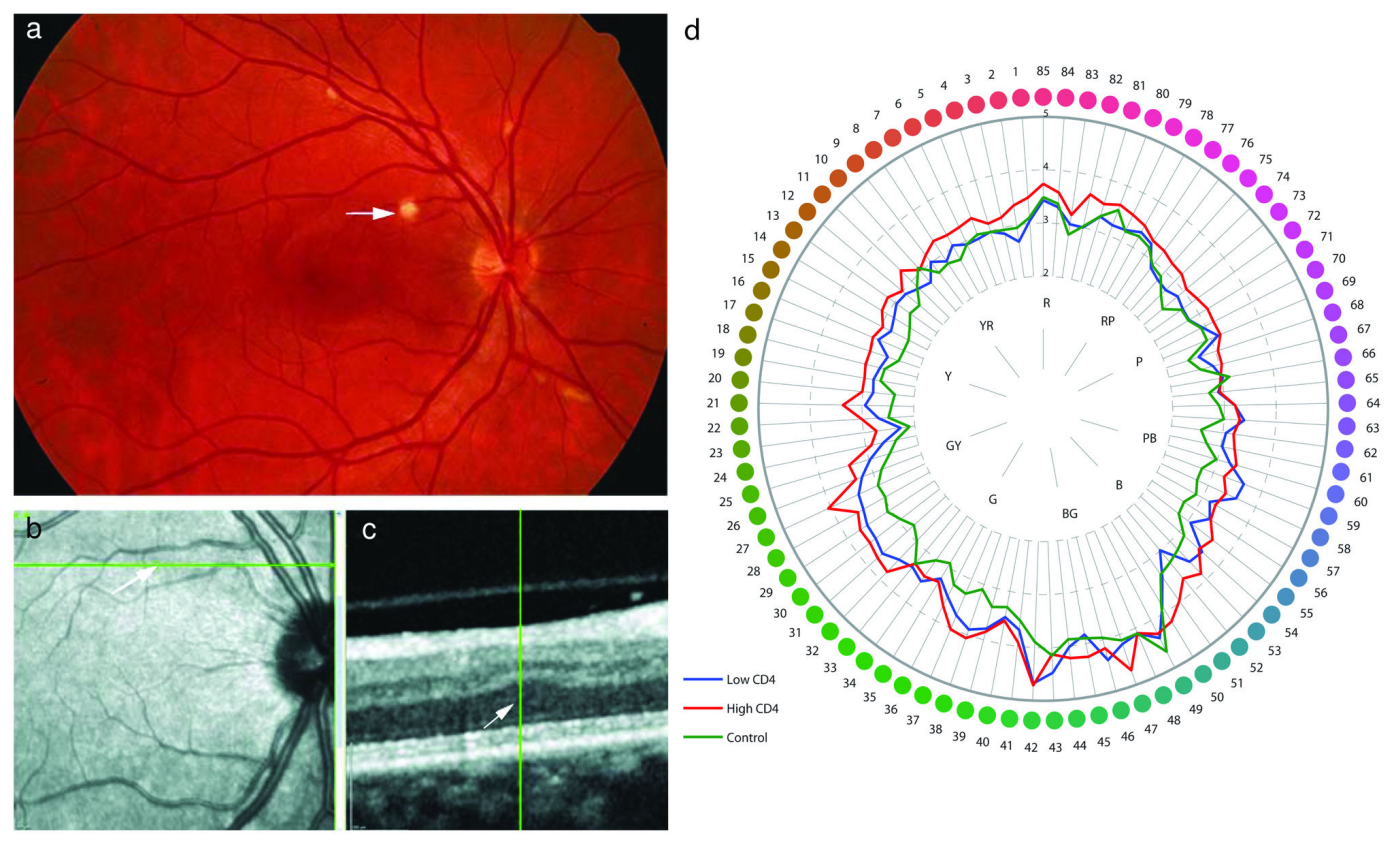
Oncotic lesions (CWS) in the retinal nerve fiber layer were investigated. After resolution permanent damage remained to the inner retinal layer. A: Color photograph of the retina of an HIV-positive patient with non-infectious retinopathy. Cotton wool spots (arrow) are present along the retinal blood vessels. B: Infrared image of the same retina 18 years after resolution of the cotton wool spot with the arrow pointing to its previous location. The green line represents high-resolution spectral-domain optical coherence tomography (SD-OCT) sectioning through the ischemic spot co-localizing it to an adjacent B-scan. C: Adjacent B-scan showing all retinal layers. The hyper-reflective layer on the top is the nerve fiber layer, original location of the cotton wool spot, which shows thinning. There is a compensatory shift of outer retinal layers including the outer nuclear layer towards the inner retina (arrowhead). D: Farnsworth-Munsell diagram showing averaged data for all three groups of eyes. Each group - the group with high CD4, the group with low CD4, and the control group – was significantly different from every other group (p < 0.0001 in each comparison).

We investigated cotton wool spots, and observed that after resolution permanent damage remained to the inner retinal layer (Figure 1 A, B). A total of 27 CWS lesions from 14 eyes of 11 HIV-positive male patients were imaged *in vivo* 2 to 18 years after the active lesion was resolved. The mean time between the documentation of the active CWS and the SLO/spectral OCT scan was 10.4 years. The median age of the patients at the time of the active retinal CWS was 37 years (range 30 to 46). All patients had CD4 T-cell counts determined within 3 months of the lesions’ appearance and were below 100x10^6^/L. Analysis of each retinal layer thickness showed that years after the resolution of a CWS there was a statistically significant thinning of the inner and middle retinal layers of the retina: the retinal nerve fiber layer (NFL), ganglion cell layer (GCL), inner nuclear layer (INL) and outer plexiform layer (OPL) (*p* < 0.001) while the outermost inner-retinal layer, the outer nuclear layer (ONL), was thickened by 17 percent (Figure 1C) (*p* < 0.001). The thickness of the ONL corresponded to an expansion of tissue towards the vitreous as a result of the loss of inner-retinal layers. The greatest tissue loss of 39% was seen in GCL, followed by the INL and OPL with 22% and 21% tissue loss respectively. There was no difference in outer retinal thickness in the center of the fovea between HIV-positive and HIV-negative human eyes (*p* = 0.18).

### Resolved CWS cause a permanent reduction in local retinal sensitivity

Analysis of resolved retinal CWS by microperimetry revealed a permanent reduction in local retinal sensitivity consistent with interruption of signal transmission from the posterior pole photoreceptors beneath these CWS to the brain. The mean microperimetric retinal sensitivity in long-term resolved CWS was (11.17 ± 4.31 s.d.) dB. The mean sensitivity of the surrounding retina controls was (14.06 ± 3.17 s.d.) dB (*p* = 0.02). When a single point of lowest sensitivity was compared to the control retina the difference was more statistically significant (*p* < 0.00001). This demonstrates that in addition to the permanent anatomical changes observed after resolution of CWS, a persistent functional deficit remained in those same areas (Figure 1C).

### Color vision is reduced in HIV-positive individuals

We demonstrated impaired visual function in our patient cohort using psychophysical tests. The Farnsworth-Munsell 100 hue test (Figure 1D) uncovered a reduction in color vision amongst HIV-positive patients with significant differences observed between patient groups stratified by CD4 count. The error scores of the control group are lower than those of the patient groups. The loss of color vision between groups was analyzed by two-way ANOVA and the difference between groups was found to be significant (F2,10370 = 72.9; *p* < 0.0001). There was also a significant main effect of Farnsworth-Munsell item number (F84,10370 = 10.4; *p* < 0.0001), meaning that error scores are significantly higher for some items than others. However, this is not group-specific: There is no significant interaction between group and Farnsworth Munsell item number (F168,10370 = 0.52; *p* ~ 1). The color vision deficit is general and not confined to a specific axis; therefore it is not due to loss or selective damage to one of the three color pathways within the retina. This finding points to a general decrease in retinal function.

### An example of the CWS microperimetric abnormality demonstrating long term-damage

**Figure 2:**
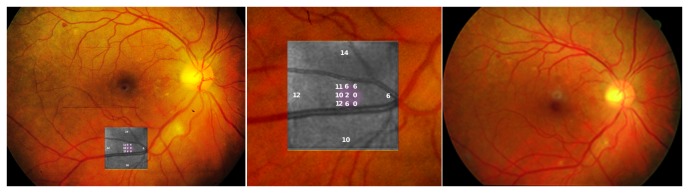
Example of the CWS microperimetric abnormality demonstrating long term-damage. Left Panel: Microperimetry examination overlaid on color fundus photograph, at the area of the previous cotton wool spot. An intraretinal hemorrhage is present superotemporal to the macula and a CWS is inferior to the optic nerve. Middle Panel: Enlarged image of microperimetry overlay showing sensitivity values in dB over the area of CWS shows decreased sensitivity (lower numbers in violet) over the area of previous cotton wool spot demonstrating long-term damage. Right Panel: Color photograph of the retina of the same eye with a dot hemorrhage superotemporal to the macula and fading cotton wool spot.

An example of this microperimetric abnormality is presented in Figure 2. The microperimetry examination is overlaid on the color fundus photograph at the area where the cotton wool spot was previously (left panel). In the example shown, an intraretinal hemorrhage is present superotemporal to the macula and a CWS is inferior to the optic nerve.

An enlarged image of microperimetry overlay is presented in the middle panel. Sensitivity values in dB are given, highlighting the decreased sensitivity over the area of previous cotton wool spot. This demonstrates the long-term damage that occurs. A color photograph of the retina of the same eye with a dot hemorrhage superotemporal to the macula and fading cotton wool spot is presented in the right panel.

### Molecular changes in HIV positive retinae revealed altered visual perception

**Figure 3:**
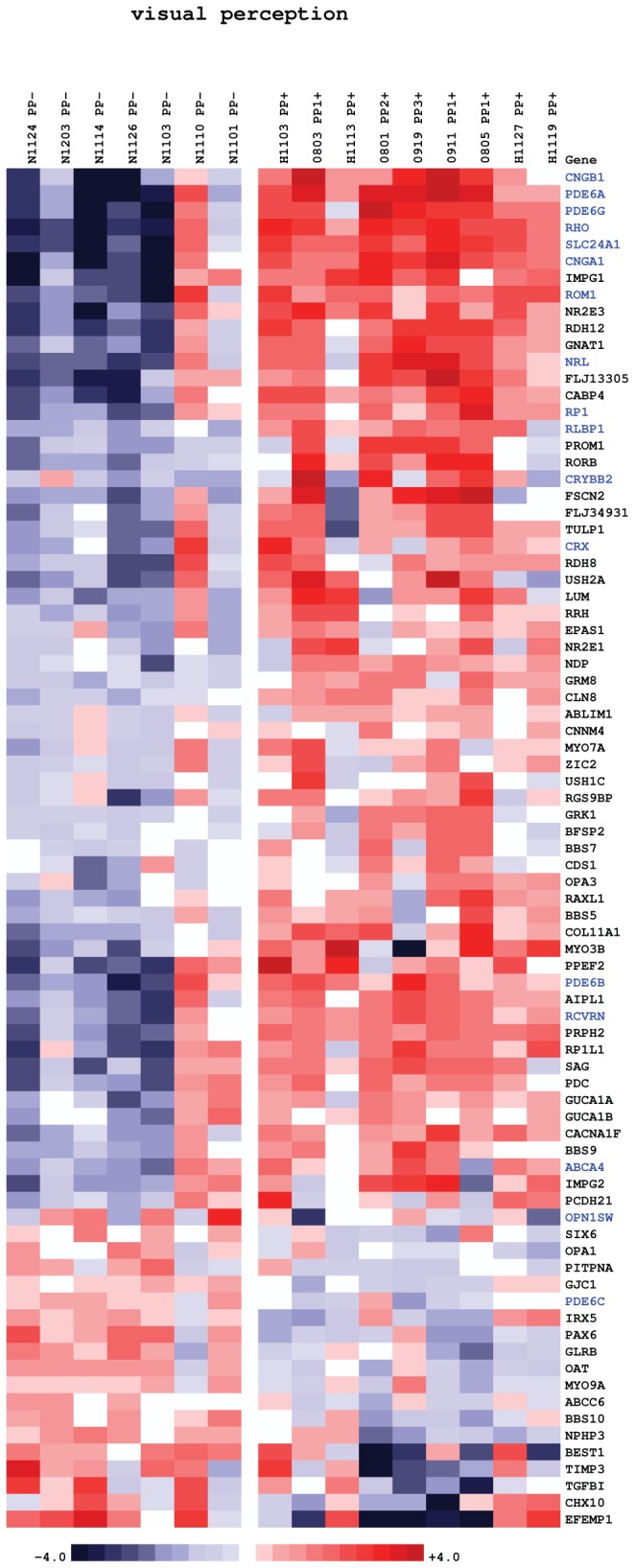
Heatmap of gene expression changes between posterior pole retinal punches of HIV-positive (10 samples on the right) and HIV-negative donor eyes (six samples on the left). Red and blue boxes indicate relative over- and under-expression with respect to a mean level between the two groups. Only genes with regularized *t*-statistic > 1 have been included. Symbols in blue indicate genes discussed in text. The samples are ordered by the donors’ time of death from left to right in each group.

**Figure 4:**
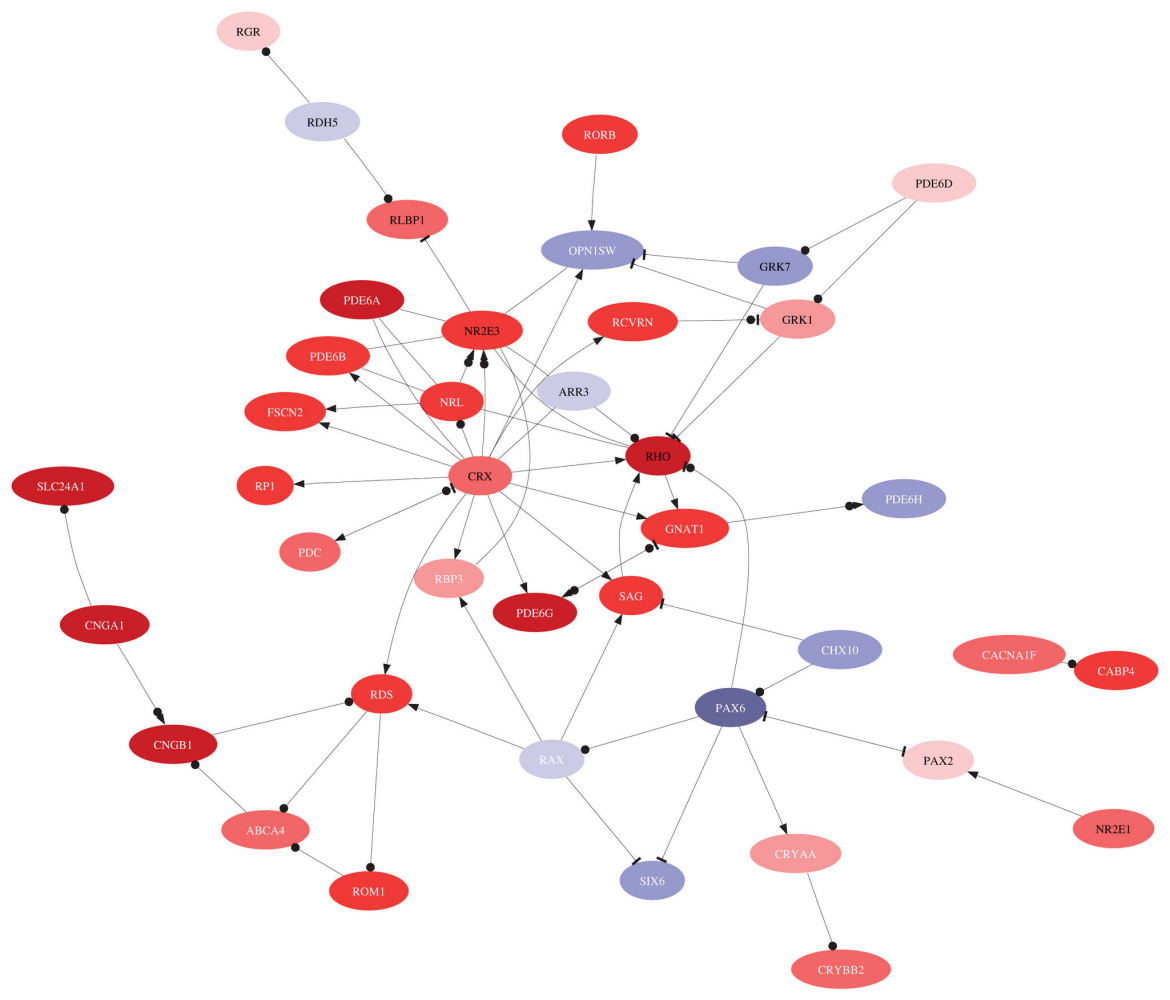
Interaction network of genes involved in visual perception using MetaCore™ knowledge base [48]. The color reflects direction and size of regulation in HIV-positive retinae compared to HIV-negative controls. A link signifies interaction, a bullet is binding of protein products, an arrow means regulation of transcription or activation, and a hammer means inhibition or deactivation.

To understand the molecular changes in these retinae, we sampled retinal tissue in HIV positive and negative patients and included areas affected by macroscopically visible retinopathy. For transcriptome analysis we extracted RNA from 27 posterior pole punches; 7 samples from 7 HIV-negative autopsy donors and 20 from 14 HIV-positive donors. The latter group consisted of 4 CWS, 6 IR hemorrhages, and 10 samples from ophthalmologically normal areas of the posterior pole. In the microarray comparison of lesion-free HIV-positive retinae with HIV-negative retinae we noted that out of 18,429 detected probes, 2004 were differentially regulated at a false discovery rate (FDR) level of 0.01, and 4,541 probes at a FDR level of 0.05. Every gene specifically mentioned in this comparison is significant at FDR < 0.05 unless otherwise stated. The biological process most significantly represented was visual perception (adjusted p-value < 10-12), (Figure S1). This process is presented in Figure 3 as a heat map to view differential transcript expression and in Figure 4 as a systems view network of interacting genes and gene products. Since retina is a heterogeneous tissue with at least five distinct neuronal and epithelial cell types, expression data is a superposition of signals from the constituent cell types. We focus on unique cellular markers, which allow us to figuratively peel the layers and glean individual components.

The salient feature of this comparison is upregulation of rhodopsin *RHO* in HIV-positive retinae (fold change f = 4.4), rod-specific phosphodiesterase-6 phototransduction enzyme (subunits *PDE6A*, *PDE6B* and *PDE6G*, f = 5.2, 2.2 and 3.8, respectively), and phototransduction enzyme *CNG4* (subunits *CNGA1* and *CNGB1*, f = 4.0 and 3.9, respectively) (Figures 3 and 4). The key photoreceptor-specific transcription factor *CRX* (f = 1.6) and its partner *NRL* (f = 3.1) were also upregulated. It should be noted however that expression of *CRX* has been found additionally in RPE [20]. Retinitis pigmentosa 1 (RP1), which encodes an enzyme involved in rod outer segment morphogenesis, was also upregulated (f = 2.3). Transcripts of photoreceptor-specific structural proteins, rim protein *ABCA4* and retinal outer segment membrane protein *ROM1*, which is essential for disk morphogenesis, were similarly upregulated (f = 1.6 and 2.8, respectively). Crystallins *CRYAA* and *CRYBB2*, which, in retina, are involved in photoreceptor outer membrane renewal [21], were both upregulated (f = 1.3, FDR < 0.2, and f = 1.8, respectively). Retinaldehyde-binding protein 1 (*RLBP1*), which is responsible for regeneration and *de novo* synthesis of retinoids that cycle between the photoreceptors and RPE was also upregulated (f = 2.0).

Collectively, upregulation of all rod OS markers, including structural components, is consistent with an increased (~2-4-fold) growth rate of rod OS. As the control and HIV donor groups were not perfectly age-matched (controls were older by an average of 21 years), the upregulation of *RHO* and other rod-specific genes could in principle be explained by a 2-4-fold loss of rods among the older control donors. However, ample evidence exists to the contrary: the total amount of *RHO* in the eye remains constant between infancy and up to 94 years of age [22]. In addition, no histological evidence exists for such a dramatic rod loss in a normally ageing geriatric population. Homeostasis demands that the faster growth of POS indicated by the transcriptomics data is matched by an increase in the rate of phagocytosis of POS by RPE. This conclusion is in *quantitative* agreement with Canki et al. [18], who observed that in culture, exposure to HIV-1 caused a two- to four-fold increase in the binding and uptake of isolated rod OS by RPE cells. Increased phagocytosis by RPE is also supported by the observed upregulation of c-mer proto-oncogene tyrosine kinase *MERTK*, a critical regulator of RPE phagocytosis (f = 1.6) [23]. RPE lacking functional *MERTK* protein is defective in phagocytosis of POS, whose accumulation leads to retinal dystrophy [24].

The increased rate of rod OS synthesis is adopted by the retina as a reaction to increased phagocytosis by RPE in order to prevent total photoreceptor and vision loss. Zhang et al. reported that, in culture, RPE cells fed by POS became overloaded with calcium and accumulated lipofuscin [25]. Excessive calcium inflow may lead to impairment of function and cell death [26]. Intracellular calcium has been linked to ageing in brain cells, which is also accompanied by lipofuscin accumulation [27,28]. We posit that increased RPE phagocytic activity induced by HIV infection leads to calcium accumulation and premature aging of the RPE layer via functional alterations in the absence of thinning or other gross morphological changes. We note that of the 39 RPE-specific genes identified in a recent screening study [29], 12 were found significantly regulated (FDR < 0.1), and all with the exception of *RLBP1* are downregulated (Figure S2). Monocarboxylate transporter 3 *SLC16A3*, whose decline correlates with advancing degeneration of RPE in AMD [30], is downregulated (f = -1.4). Likewise, 7-dehydrocholesterol reductase *DHCR7*, whose inhibition in an animal model leads to RPE degeneration [31] is downregulated (f = -1.3).

### Cone-specific transcripts were moderately yet systematically downregulated in HIV positive retinae

The posterior pole samples in this study were taken from a region 3-4 mm from the fovea and contained an estimated 5-6% of cones, 94-95% of rods. Partial cone loss would not therefore result in gross morphological changes but rather manifest as a downregulation of cone-specific transcripts, including opsins. This scenario is consistent with our findings: cone-specific genes were moderately yet systematically downregulated: Most notably, opsins *OPN1SW* (f = -1.4) and *OPN1LW* (f = -1.4, FDR = 0.07), cone-specific *PDE6* (subunits *PDE6C,* f = -1.3, and *PDE6H*, f = -1.4, FDR = 0.15), and *GRK7* (f = -1.2, FDR = 0.13), an enzyme with high specificity to cone opsins (Figures 2 and 3).

The exact nature in which the ageing RPE affects rods and cones differentially remains to be elucidated, but it is certainly logical from an evolutionary viewpoint since color vision evolved as a modification of and augmentation to an already present monochromatic vision (in Arthropoda) [32], thus one would expect rods to survive adverse conditions better than cones. Selective cone death has been observed in genetic retinopathies induced by genetic manipulation of RPE layer [33] but also the ganglion cell layer [34]. Aside from obvious morphological differences, there has to be a fundamental molecular-level difference between rods and cones that renders cones more susceptible to cell death. One likely possibility is the different manner in which rods and cones manage calcium signaling and homeostasis [35]. Genes involved in rod calcium signaling and homeostasis, recoverin *RCVRN* (f = 2.3) and Na-Ca+K exchanger *SLC24A1* (f = 4.0) were significantly upregulated. Retinal cone Na-Ca+K exchanger *SLC24A2* is unchanged. The Intracellular Calcium Signaling pathway as a whole was significantly altered in HIV retinae (adjusted *p*-value = 0.001) (Figure S3).

### Comparison of tissue with an active CWS with lesion-free retina

In order to compare tissue with an active CWS with lesion-free retina, we compared 4 CWS samples with 4 HIV-positive but otherwise ophthalmologically normal controls. 17 genes were called differentially expressed at FDR level of 0.1. Amongst the relevant biological processes, *inflammatory response* (adjusted *p*-value 10^-9^) was prominent. However, pro-inflammatory microglia-stimulating signals are downregulated in CWS: allograft inflammatory factor 1 *AIF1* (f = -1.8), S100 calcium binding proteins *A8* and *A9* (f = -2.4 and -2.7) (Figure S4).

### Comparison of tissue with IRH to lesion-free retinae

In order to compare tissues with IRH to lesion-free retinae, we compared 6 samples with IRH to 6 samples of control HIV-positive retinae. Four genes were differentially regulated at the FDR level of 0.1. Thrombospondin 4 (*THBS4*), a modulator of endothelial cell proliferation expressed by vascular cells is upregulated (f = 1.6), which suggests rebuilding of the vascular system at the site of injury. As in the case with CWS, it appears that IRH lesions do not cause any additional modification to the outer retina (Figure S5).

### Examination of the influence of diurnal rhythm

**Figure 5:**
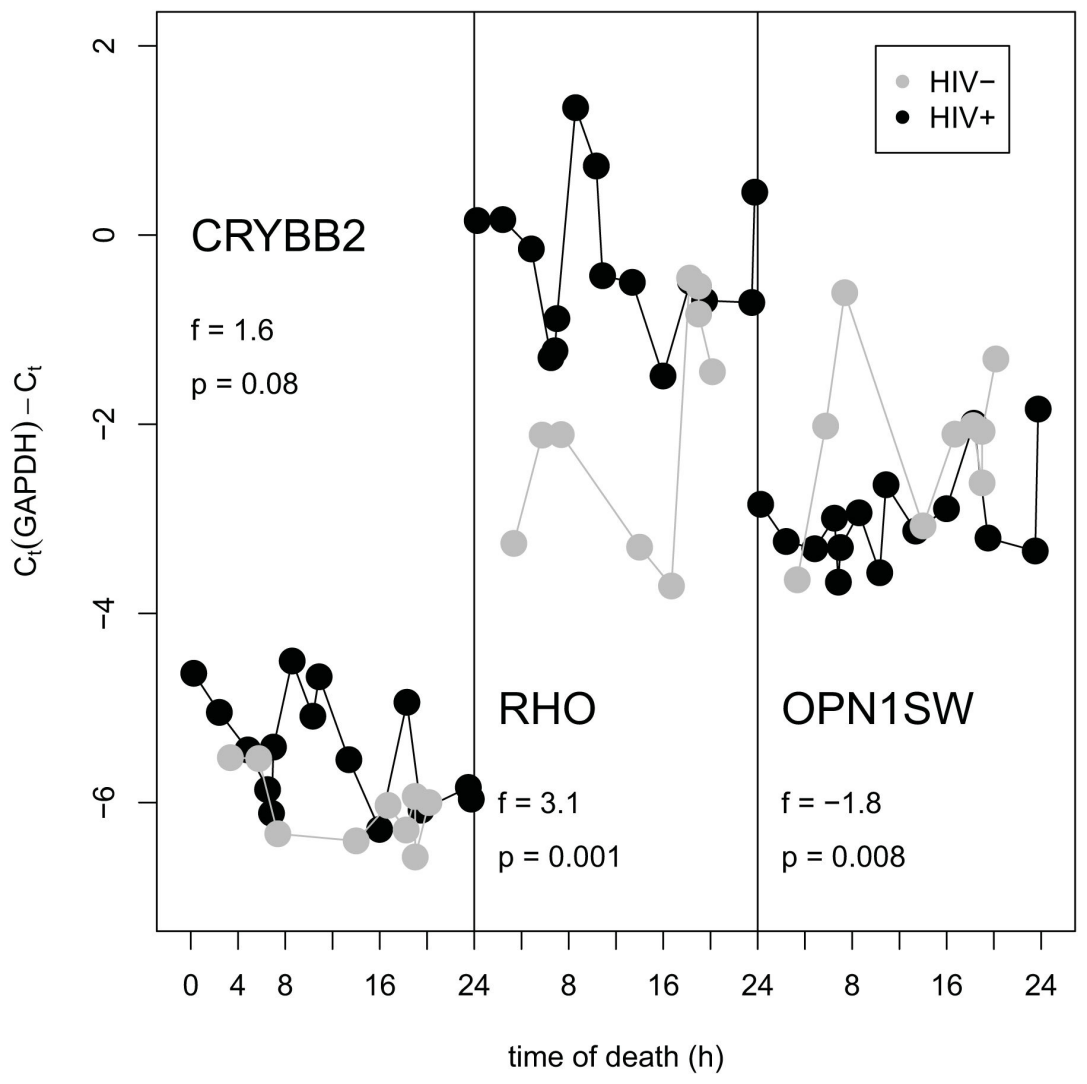
Abundance of transcript measured by qPCR (relative to a reference gene *GAPDH*) in retinae of HIV-negative and HIV-positive donors as function of time of death.

There is one other possible explanation for the reported molecular differences between HIV-negative and HIV-positive retinae: It is conceivable that the retinal samples were harvested in such a manner that the majority of the HIV-negative samples originated from a different time of day (TOD) than most of the HIV-positive samples. This would confound the analysis and the detected differences could primarily be due to diurnal rhythm rather than HIV status. In order to verify our microarray results and to investigate a potential role of diurnal regulation on some of the transcripts, we performed qPCR measurements of abundance of three transcripts, *RHO*, *OPN1SW* and *CRYBB2*, for 15 HIV-positive retinae and 9 HIV-negative controls. Figure 5 is a plot of transcript abundance (relative to a reference gene, *GAPDH*) as a function of TOD. The observed fold inductions are in a quantitative agreement with microarray results. No obvious diurnal rhythm was observed with any of these transcripts.

Our study is the first to show significant outer retinal dysfunction in HIV-positive retinae. There has been much evidence to date that inner retinal damage exists in HIV-positive patients and that this affects structure and function but these effects are mild to moderate in severity. It is known that inner retinal disease can affect the outer retina in eyes with inner retinal damage due to optic neuropathy [36]. It has also been demonstrated that there is inner retinal disease in patients with advanced outer retinal degeneration [37-39]. In animal models of outer retinal dystrophy, as the outer retinal degeneration progresses there is inner retinal disorganization as well [40,41]. For these reasons we cannot state definitively whether the distinct photoreceptor dysregulation we have documented is independent of the known inner retinal damage in HIV or whether the photoreceptor problems we outline in this study contribute to the inner retinal damage.

## Conclusions

In summary the systems wide studies reported here indicate dysfunction retinal pathways unique to the phototransduction process, i.e. rods and cones. This is the first report of a previously unrecognized source of vision loss, not accounted for by inner retinal disease (CWS, superficial retinal hemorrhages and capillary drop out). This photoreceptor damage/dysfunction is not associated with thinning or loss of outer retinal layers as evidenced by spectral domain optical coherence tomography (SD-OCT). Additionally we uncovered modulation of inflammatory response in areas affected by active CWS and changes in several vascular marker pathways in areas with hemorrhages. This suggests that the CWS do affect the inner retina but not the outer retina, but both retinal layers are damaged in HIV infection.

## Materials and Methods

### Ethics Statement

The study was approved by the University of California San Diego Institutional Review Board (http://www.sandiego.edu/irb), and informed consent was taken from all study patients. Study participants provided written informed consent to participate in this study. The consent procedure was approved by the UCSD Institutional Review Board ethics committee. Both the HIV-positive patients and non-HIV age-matched healthy participants come from a UCSD Institutional Review Board-approved, National Institutes of Health-sponsored longitudinal study of HIV disease at the University of California, San Diego (UCSD). The research followed the tenets of the Declaration of Helsinki.

### Structural evaluation *in vivo* of the retina after ophthalmoscopic resolution of cotton wool spots

We performed a retrospective study of HIV patients who were determined by clinical examination and fundus photographs to have had retinal cotton wool spots (CWS) at least 2 years prior to the initiation of the study. The clinically active CWS disappear within 4 to 12 weeks leaving a localized area of ischemic atrophy in pathology reports. All CWS were confirmed by examination of stereo color transparency slides taken with a color fundus camera (Canon USA, Lake Success, NY or Topcon Incorporated, Paramus, New Jersey) and recorded on ASA 100 35 mm film. Patients were imaged with a simultaneous scanning laser ophthalmoscopy (SLO)/ Spectral OCT (optical coherence tomography) device using red free and infrared SLO imaging to portray fundus landmarks including vessels (Heidelberg Spectralis OCT, Heidelberg Engineering, Vista, CA). The serial scans were co-localized to the area of the initial retinal cotton wool spot using the overlay software on the OCT. The color fundus photograph and the SLO image were registered and superimposed on each other using commercial imaging software (Adobe Photoshop). The scan line and point along that line closest to the center of the ophthalmoscopically visible cotton wool spot was the one chosen for the analysis and quantification of retinal layer thickness (Figure 1).

Each CWS lesion had a control area taken from the same eye. That area was chosen from the adjacent retina in an area free of vessels within 1-2 disc diameters from the cotton wool spot and approximately along the same nerve fiber layer arc. Measurements of each retinal layer were done in microns by two observers who were kept masked to whether the lesion was a control or a resolved CWS. Each retinal layer was measured in microns and then the data recorded for statistical analysis. The thickness of each of the layers of retina at the cotton wool spot was compared to its corresponding layers at the nearest control area using the paired t-test. The SAS (SAS Institute Inc., NC; version 9.2) multi-test procedure was used to adjust the p-values from a family of hypothesis tests. The Benjamini and Hochberg False discovery rate (FDR) control method was used to control for multiple retinal layer measurements per lesion.

### Assessment of retinal function in areas of resolved CWS by microperimetry

In order to further document the permanent anatomical changes of long term resolved cotton wool spots just described and to evaluate if these changes translate into a permanent deficit in retinal sensitivity, we performed microperimetry (OPKO instrumentation/OTI, Miami FL). During a microperimetry test, a subjective measurement of the visual function in a relatively small area of their retina (10° to 20°) is obtained. A patient is shown visual stimuli at specific light intensities, at specific locations on their retina. The patient uses a hand held button/clicker to notify the system if they saw the stimulus. That feedback (or lack thereof) determines the next intensity to present that stimulus at. This process is repeated for all of the stimuli in a predetermined pattern and predetermined area. At the end of the test the operator is given a fundus image with the stimulus pattern overlaid showing the dimmest intensity at which each stimulus was seen by the patient. The intensity level of the stimulus is displayed in dB. The test was performed in the exact location of the previous CWS and in normal adjacent retina in the same eye for control.

We used a custom designed pattern that allowed us to test the retinal sensitivity in the area of the previous CWS and the control area at the same time to avoid patient fatigue. It consisted of 6 testing points in the area of the resolved CWS and 4 adjoining points located 950 microns from the center of the grid to be used as nearby controls. The size of the stimulus used was 400 minarc^2^ (Goldmann III), with an area of 9156 square microns, 108 micron as retina spot size diameter with a 150msec duration which was sufficiently small to detect a lesion and brief enough to avoid triggering eye movements. The starting stimulus light attenuation was set at 10 dB.

The fixation target used was a red or green circle depending on the color each patient preferred. To compensate for the learning curve, a 2x2 grid test pattern was done in each eye prior to the testing. The color fundus photograph containing the original CWS and the microperimetry map image obtained were superimposed on each other using commercial imaging software (Adobe Photoshop).

The average of the microperimetry points within the CWS was obtained as the lesion’s retinal sensitivity. The average of the 4 adjoining dots was used as control. Any test spot overlying a vessel was discarded as blood vessel reduced retinal sensitivity by microperimetry. For statistical analysis, we used a paired t-test, JUMP version 8.0 (SAS inc. Carey, North Carolina)

### Examination of color vision in HIV eyes

#### Patients

For color vision function we compared performance between HIV-positive and control subjects. Both the HIV-positive patients and non-HIV age-matched healthy participants for this assessment and the ones mentioned above come from an Institutional Review Board-approved, National Institutes of Health-sponsored longitudinal study of HIV disease at the University of California, San Diego (UCSD). The research followed the tenets of the Declaration of Helsinki.

The patients were divided into three groups. The high CD4 group consisted of HIV-positive patients with good immune status whose CD4 counts were never valued at <100 (1.0x10^9^/L). The low CD4 group were HIV-positive patients with CD4 cell counts measured at <100 (1.0x10^9^/L) at some period of time in their medical history. None of the eyes had evidence of retinopathy of other etiology. All HIV patients were on combination antiretroviral therapy (cART) therapy prior to the time of the examination and a substantial portion of these patients had a recovery in their CD4 counts. The HIV individuals had no confounding ocular disease or eye surgery. The normal group consisted of HIV- negative patients without evidence of ocular damage.

#### Ophthalmologic evaluation

All patients had a complete ocular examination, including indirect ophthalmoscopy. The exclusion criteria were corrected visual acuity worse than 20/40, spherical refraction beyond ± 5 diopters, cylindrical correction greater than 3 diopters, unclear ocular media, concurrent or healed CMV retinitis (a fellow eye without retinitis was eligible), scotopic pupil size < 3mm, glaucoma or suspicion of glaucoma, and diseases that can cause retinopathy, like diabetes or uncontrolled hypertension.

#### Color vision hue discrimination testing

We performed Farmsworth-Munsell 100 hue discrimination test (FMTS, Velbert, Germany) with computerized records. The second test of each participant was analyzed to eliminate practice effect. The raw error score (Windows-based PC scoring software included) for each eye (f=85) represented the difference at each position of the color circle of the expected cap and the selected cap. This set of values was statistically compared (Tukey HSD post-hoc pair-wise comparisons) among three groups.

### Tissue procurement

The autopsy eyes were procured from patients who had died of clinically diagnosed AIDS. The eyes from both HIV-positive and HIV-negative donors were obtained from either the San Diego Eye Bank, the National Disease Research Institute or the California NeuroAIDS Tissue Network including the University of California San Diego (UCSD) autopsy service.

To prepare the total mRNA, eyes were opened at the limbus post-enucleation (< 24 hrs post mortem), immediately placed in 50 mL of RNAlater (Ambion Inc., Austin, TX), and transported on ice to our laboratory at the Shiley Eye Center, University of California at San Diego (La Jolla, CA). In order to assure prompt delivery of unfixed and fresh ocular material, a diener was paid to enucleate the eyes, properly immerse them in RNAlater, and then deliver them to the laboratory, where they were placed at 4°C until dissection (up to 7 days). All work was conducted employing Biosafety Level-II standards and practices.

### Dissection

Upon arrival to our lab, received eyes were recorded on a data record with available patient information. Each eye was identified, accessioned, and measured. At gross examination, the limbal incision was enlarged, and the cornea and lens removed, allowing dissection of lesions under a dissecting microscope. After the remaining anterior segments (vitreous humor) and excess RNAlater were partially removed, the eye was examined. Gross pathological findings were recorded both by photograph and on an autopsy data sheet. The remaining vitreous was then gently and completely removed, and 4 radial peripheral retinal incisions were made, while avoiding the macula and major blood vessels, in order to lay the eyecup flat. Separate, PCR clean, 2 mm trephines were used to punch individual retina in areas of posterior pole with visible cotton wool spots (CWS) or intraretinal hemorrhages (IH). If absent the punches were taken from the retina in the posterior pole. Six control punches from the peripheral retina were also taken from each eye. The position of each biopsy was recorded on the autopsy data sheet. Each punch was placed into a separate pre-labeled vial containing 1.0 mL TRIzol reagent (Invitrogen Life Technologies, Carlsbad, CA), snap frozen (-80°C) and stored until processing and individual analysis. Eyes containing CMV retinitis or retinitis lesions were excluded from this study.

### Isolation of total RNA for microarray studies

Retinal biopsies were placed directly in 1 mL TRIzol reagent prior to nucleic acid extraction. The TRIzol protocol was followed exactly as recommended by the manufacturer (Invitrogen, Carlsbad, CA). Briefly, the biopsies were needle-sheared, total RNA precipitated, purified and air-dried. The pellet was then resuspended in a solution of water, DNase buffer and DNase enzyme (Qiagen, Valencia, CA), and incubated at room temperature 10 minutes to remove any genomic DNA contamination. Total RNA was then purified on a Qiagen RNeasy column according to manufacturer’s instructions. Elution was carried out with RNase/DNase-free water in two parts, combined and concentrated using a Savant Integrated SpeedVac Vacuum System until the total volume reached 15 µL. For RNA quantitation, the NanoDrop ND-1000 (NanoDrop Technologies, Inc. Wilmington, DE) was used. This instrument measures the absorbance of RNA at 260 nm from 1 µL of sample. The samples were then transported to the UCSD BIOGEM core for Illumina Beadarray processing.

### DNA Microarray Experiments

Biotinylated cRNA was prepared using the Illumina RNA Amplification Kit, Catalog #1L1791 (Ambion, Inc., Austin, TX) according to the manufacturer’s directions starting with 250 ng total RNA. For microarray analysis, the Illumina Human 6 Sentrix and HumanHT-12 v4 Expression BeadChip Kits were used (Illumina, San Diego). Hybridization of labeled cRNA to the BeadChip, and washing and scanning were performed according to the Illumina BeadStation 500x manual. Essentially the amplified, biotin-labeled human cRNA samples were resuspended in a solution of Hyb E1 buffer (Illumina) and 25% (v/v) formamide at a final concentration of 25 ng/µL. 1.5 µg of each cRNA were hybridized. Hybridization was allowed to proceed at 55°C, for 18 hours after which, the bead array matrix was washed for 10 minutes with 1X High temperature buffer (Illumina), followed by a subsequent 10 minute wash in Wash E1BC buffer. The arrays were then washed with 100% ethanol for 10 min to strip off any remaining adhesive on the chip. A 2 minute E1BC wash was performed to remove residual ethanol. The arrays were blocked for 5 minutes with 1% (w/v) casein-PBS, (Pierce). The array signal was developed via 10 minute incubation with Streptavidin-Cy3 at a final concentration of 1µg/mL solution of (GE Healthcare) in 1% casein-PBS blocking solution. The Expression BeadChip was washed a final time in Wash E1BC buffer for five minutes and subsequently dried via centrifugation for 4 minutes at a setting of 275 rcf.

The arrays were scanned on the Illumina BeadArray Reader, a confocal-type imaging system with 532 (cye3) nm laser illuminations. Image analysis and data extraction was carried out as in accordance with Illumina specifications. Preliminary data analysis and QC was carried out using the GenomeStudio software (Illumina). All array data has been deposited in the EBI ArrayExpress Database. The ArrayExpress accessions are E-MEXP-3758 and E-MEXP-3757

### Analysis of microarray data

#### 1) Normalization of microarray data

Expression level data from the Illumina Bead Studio software were normalized using a multiple-*loess* algorithm [42]. Probes whose expression level exceeds a threshold value in at least one sample are called detected. The threshold value is found by inspection from the distribution plots of (log) expression levels.

#### 2) Sorting the probes according to significance

Detected probes are sorted according to their *q*-value, which is the smallest false discovery rate (FDR) at which the gene is called significant. FDR is the expected fraction of false positive tests among significant tests [43]. We evaluate FDR using Significance Analysis of Microarrays (SAM) and its implementation in the official statistical package *samr* [44]. In order to not be unduly impressed by accidentally small variances, we set the percentile of standard deviation values used for the exchangeability factor *s0* in the test statistic to 75.

#### 3) Statistical analysis of pathways and gene ontology terms

Each gene ontology term or a pathway is treated simply as a set of genes. The probe list, sorted by *q*-value in ascending order, is translated into Entrez gene ID’s and parsed so that whenever several different probes represent the same gene, only the highest-ranking probe is kept for further analysis. The sorted list of genes is subjected to a non-parametric variant of the Gene Set Enrichment Analysis (GSEA) [45], in which the *p*-value of a gene set of size *n* is defined as follows: Let us denote the *k-*th highest rank in gene set as *r*_*k*_, and define *p*_*k*_ as the probability that out of *n* randomly chosen ranks (without replacement) the *k-*th highest is not smaller than *r*_*k*_. The p-value of the gene set is defined as min_*k*_ [*P*_*k*_] It is designed to detect overrepresented gene sets at the top of the list. Unlike the Kolmogorov-Smirnov statistic used in GSEA, it will not detect underrepresented or other, pathologically distributed, gene sets. Finding the *p*-value of a gene set of size *n* requires calculation of *n* rank-order values *p*_*k*_, however, there is no need to adjust the *p*-values for multiple testing as the rank-order tests are highly statistically dependent. We do perform a Bonferroni adjustment of gene *set p*-values for the number of gene *sets* tested, even though there are often several gene sets with overlapping gene content (and therefore are statistically dependent), which is partly due to the design of the gene ontology database and partly because genes tend to be involved in multiple processes. We report only gene sets with adjusted *p*-values ≤ 0.01. Because of the above-mentioned overlap of gene membership among gene ontology terms, we believe that the significant terms should not be reported as independent gene sets. Instead, we cluster the significant gene sets using variation of information (VI) as the distance metric [46], and present them graphically in this context. VI between two gene sets is also called shared information, and is a true metric. Gene sets that cluster together generally share a number of member genes.

Heatmaps of expression levels were created using in-house hierarchical clustering software. The colors qualitatively correspond to fold changes with respect to a reference which is calculated as the mid-point between compared groups.

### Q-PCR Analysis

In order to investigate the influence of diurnal rhythm as an explanation for the reported molecular differences between HIV-negative and HIV-positive retinas, we employed Q-PCR. We utilized the time of death (TOD) of our subjects as a proxy for the time of day. The median TOD and standard deviation among the HIV-negative group were 12: 30PM ± 6.8h; those of the HIV-positive group 11: 00AM ± 7.1h, and the corresponding ranges were 3:21AM―8:10PM and 0:15AM―7:30PM, respectively. In order to verify the microarray results and better investigate a potential role of diurnal regulation on some of the key transcripts, we performed qPCR measurements of abundance of three transcripts, *RHO*, *OPN1SW* and *CRYBB2*, for 15 HIV-positive retinas and 9 HIV-negative controls. Data were presented as a plot of transcript abundance (relative to a reference gene, GAPDH) as a function of TOD. Q-PCR was carried out as described previously [47]

## Supporting Information

Figure S1 **Information clustering of significant (Bonferroni adjusted *p*-value < 0.01) biological processes in the comparison of HIV-negative and HIV-positive donors.** Numbers in parentheses are number of expressed genes in the process and adjusted *p*-value. Hue of the red color is proportional to (log) *p*-value; darker color means higher significance.
(PDF)

Figure S2 **Gene expression of RPE-specific genes in HIV-negative (left panel) and HIV-positive donors.**
(PDF)

Figure S3 **Calcium signaling pathway.** Gene expression in HIV-negative (left panel) and HIV-positive donors.
(PDF)

Figure S4 **Gene expression in HIV-positive samples with CWS (right panel) compared to ophthalmologically normal HIV-positive samples.**
(PDF)

Figure S5 **Gene expression in HIV-positive samples with IRH (right panel) compared to ophthalmologically normal HIV-positive samples.**
(PDF)
