# Supplementary material for: A Degenerative Retinal Process in HIV-Associated Non-Infectious Retinopathy
Source: PLoS One. 2013 Sep 17;8(9):e74712. doi: 10.1371/journal.pone.0074712 (PMC3775801; doi:10.1371/journal.pone.0074712)
Supplement: Figure S1 — Information clustering of significant (Bonferroni adjusted p-value < 0.01) biological processes in the comparison of HIV-negative and HIV-positive donors. Numbers in parentheses are number of expressed genes in the process and adjusted p-value. Hue of the red color is proportional to (log) p-value; darker color means higher significance. (PDF) [file pone.0074712.s001.pdf]

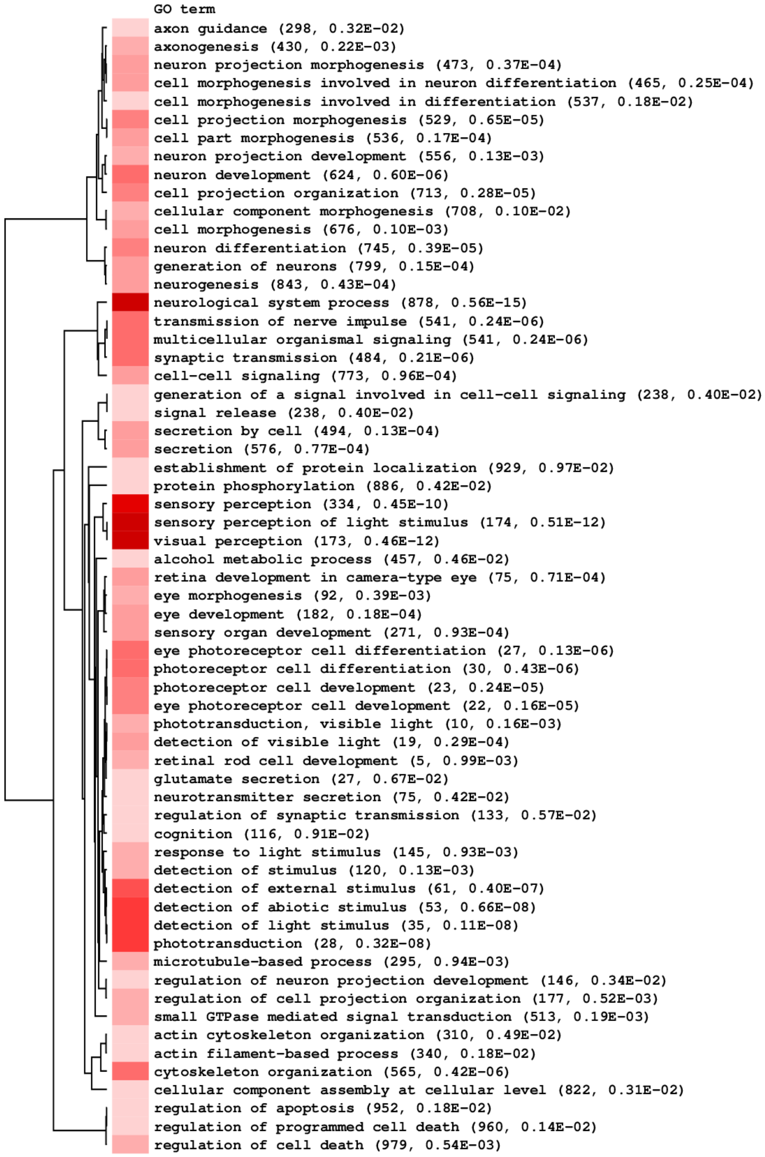

Figure S1

Information clustering of significant (Bonferroni adjusted *p*-value < 0.01) biological processes in the comparison of HIV-negative and HIV-positive donors. Numbers in parentheses are number of expressed genes in the process and adjusted *p*-value. Hue of the red color is proportional to (log) *p*-value; darker color means higher significance.
