# Supplementary material for: A Degenerative Retinal Process in HIV-Associated Non-Infectious Retinopathy
Source: PLoS One. 2013 Sep 17;8(9):e74712. doi: 10.1371/journal.pone.0074712 (PMC3775801; doi:10.1371/journal.pone.0074712)
Supplement: Figure S4 — Gene expression in HIV-positive samples with CWS (right panel) compared to ophthalmologically normal HIV-positive samples. (PDF) [file pone.0074712.s004.pdf]

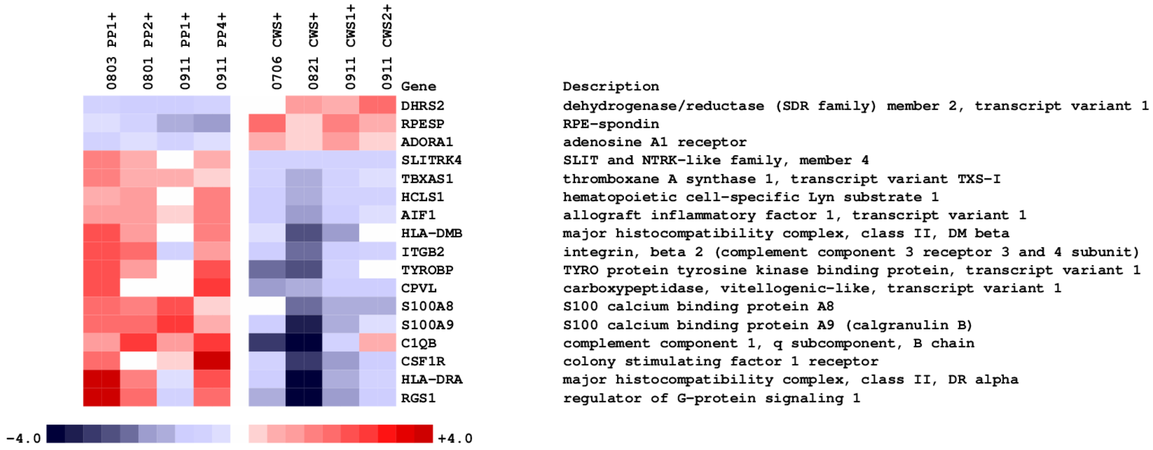

Figure S4

Gene expression in HIV-positive samples with CWS (right panel) compared to ophthalmologically normal HIV-positive samples.
