# Supplementary material for: A Degenerative Retinal Process in HIV-Associated Non-Infectious Retinopathy
Source: PLoS One. 2013 Sep 17;8(9):e74712. doi: 10.1371/journal.pone.0074712 (PMC3775801; doi:10.1371/journal.pone.0074712)
Supplement: Figure S3 — Calcium signaling pathway. Gene expression in HIV-negative (left panel) and HIV-positive donors. (PDF) [file pone.0074712.s003.pdf]

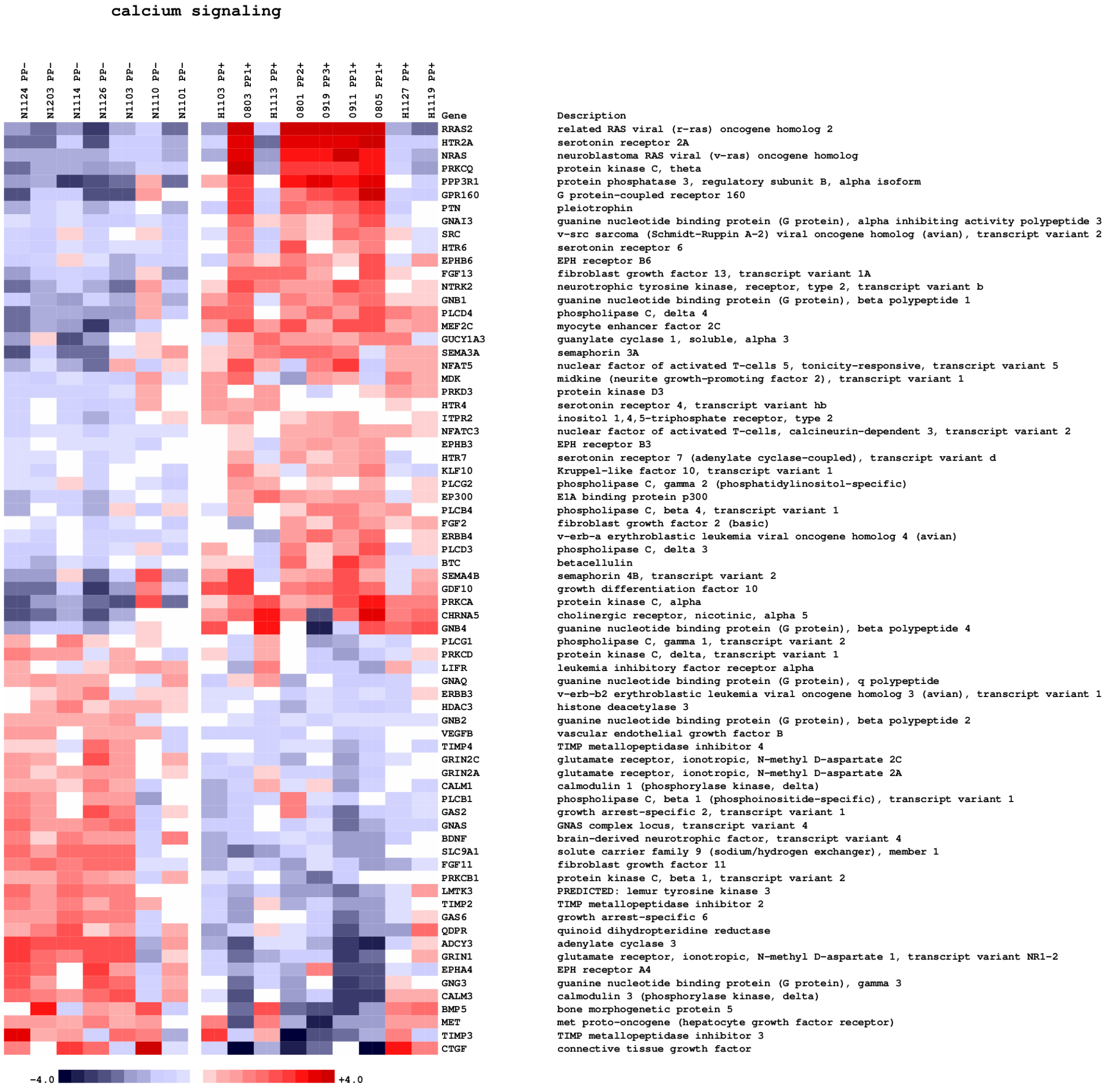

Figure S3

Calcium signaling pathway. Gene expression in HIV-negative (left panel) and HIV-positive donors.
